# Significant PTSD and Other Mental Health Effects Present 18 Months After the Fort Mcmurray Wildfire: Findings From 3,070 Grades 7–12 Students

**DOI:** 10.3389/fpsyt.2019.00623

**Published:** 2019-08-30

**Authors:** Matthew R. G. Brown, Vincent Agyapong, Andrew J. Greenshaw, Ivor Cribben, Pamela Brett-MacLean, Julie Drolet, Caroline McDonald-Harker, Joy Omeje, Monica Mankowsi, Shannon Noble, Deborah T. Kitching, Peter H. Silverstone

**Affiliations:** ^1^Department of Computing Science, University of Alberta, Edmonton, AB, Canada; ^2^Department of Psychiatry, University of Alberta, Edmonton, AB, Canada; ^3^Alberta School of Business, University of Alberta, Edmonton, AB, Canada; ^4^Faculty of Social Work, University of Calgary, Calgary, AB, Canada; ^5^Department of Sociology and Anthropology, Mount Royal University, Calgary, AB, Canada; ^6^Fort McMurray Catholic School District, Fort McMurray, AB, Canada; ^7^Fort McMurray Public School District, Fort McMurray, AB, Canada

**Keywords:** youth, mental health, wildfire, disaster, post-traumatic stress disorder, depression, anxiety, substance use and misuse

## Abstract

**Background:** The May 2016 wildfire in Fort McMurray, Alberta, Canada forced evacuation of the population of 88,000 individuals and destroyed 10% of the homes. Youth are particularly impacted by disaster.

**Methods:** Eighteen months after the wildfire, Fort McMurray Public and Catholic Schools surveyed 3,252 of the 4,407 students in Grades 7–12 to determine possible long-term psychological impacts. The survey included validated measurement scales for post-traumatic stress disorder (PTSD), depression, anxiety, use of drugs, alcohol, and tobacco, quality of life, self-esteem, and resilience. Data analysis was possible for only 3,070 students, i.e., 70% of the total student population. Anonymized data were analyzed to compare students who directly experienced lesser or greater impact from the wildfire, with greater impact defined as personally seeing the fire or having one’s home destroyed. Also, students with greater or lesser scores on the Child and Youth Resilience Measure (CYRM-12) were compared.

**Results:** Of the 3,070 students, 37% met criteria for probable PTSD; 31% met criteria for probable depression, and 17% for probable depression of at least moderate severity; 27% of students met criteria for probable anxiety, and 15% for probable alcohol or substance use disorder; 46% of all students met criteria for one or more probable diagnosis of PTSD, depression, anxiety, or alcohol/substance abuse, and this included students who were both present and not present in Fort McMurray at the time of the wildfire. Students with greater impact from the wildfire exhibited significantly higher scores on measures of PTSD, depression, anxiety, and alcohol/substance use. They also had lower self-esteem and quality of life scores. Students with lower resilience scores exhibited a similar pattern.

**Conclusions:** These findings highlight first the negative impact of disasters on youth mental health, particularly for those who directly experience wildfire, and second the role of resilience on youth mental health, with lower resilience associated with substantially lower mental health outcomes. These results emphasize the need for long-term mental health supports for youth post-disaster, with specific focus on increasing youth resilience, which may serve as a protective factor against effects of disaster on mental health.

## Introduction

On May 3, 2016, a large wildfire, called “The Beast” in the popular media (1), forced the population of 88,000 living in Fort McMurray, Alberta, Canada to evacuate. The fire destroyed 10% of the homes in Fort McMurray and burned 590,000 hectares of land before being contained. The Insurance Bureau of Canada indicated that the cost of the Fort McMurray wildfire was estimated at $3.6 billion, making this the most expensive insured catastrophe in Canadian history (2). After evacuation, many individuals were displaced because their homes were damaged or made otherwise unfit for habitation by the fire. Many individuals also faced job loss and unemployment due to the damage and closure of local businesses. In addition to physical damage to local infrastructure, the community was affected by social, emotional, and psychological difficulties that are often present after a disaster (3). Youth in particular are affected by disasters given their dependence on adults, structural vulnerabilities, as well as physical, psychological, and social factors related to the youth developmental stage. However, little research has examined the impact of disasters on the mental health and well-being of youth.

Wildfires have a negative impact on the mental health of local residents in the affected areas, as indicated by previous studies and lived experience. Studies looking at both adults and children have reported that both groups exhibit an increased incidence of post-traumatic stress disorder (PTSD) (4) and increased symptoms of depression and stress (5). Studies focusing specifically on adults indicate that adults exhibit increased incidence of PTSD (6, 7), increased symptoms of depression and anxiety (7, 8), increased levels of psychological distress (9), and increased consumption of anxiolytics hypnotics (10). Studies of children and youth show that these groups exhibit increased incidence of PTSD and depression (11, 12).

In a broader context, non-wildfire disasters, including floods, earthquakes, and tsunamis, also have an adverse effect on mental health [see review in Goldmann and Galea (13) and Kar (14)]. Disasters are thought to cause increased incidence of PTSD, major depressive disorder, generalized anxiety disorder, and substance use disorder in children and adults. Tang et al. (15) also suggest an association between disasters and increased depression. Earthquakes have also been linked with increased incidence of PTSD (16). Some studies report an increase in suicide months or years after natural disasters [reviewed in Ref. (17)]. These patterns are particularly concerning because an individual’s ability or lack of ability to cope with adversity during and after a disaster plays an important role in determining long-term mental health outcomes.

Despite mental health and well-being challenges resulting from disasters, resilience plays an important role in the lives of children and youth by serving as a protective factor that mitigates the effects of disaster on mental health. Resilience refers to an individual’s capacity to handle adverse life experiences based on their mindset, resourcefulness, and support from family, friends, and community (18, 19). Higher resilience, in the form of reliance on family and friends, has been associated with buffering of the negative effects of wildfire (9, 20). Characteristics associated with non-resilience, including low socio-economic status, low social support, and poor relationships, have been linked with greater risk of psychopathology post-disaster (13, 21). Conversely, pro-resilience characteristics such as self-efficacy, optimism, hardiness, higher perceived ability to cope, and flexible adaptive responses are associated with reduced psychological symptoms after disasters (13). This is particularly important given that children and youth have been identified as being more vulnerable to psychopathology after disasters (13).

To our knowledge, only one previous study prior to 2019 examined the impact of wildfires on youth mental health at a population level, focusing on PTSD and depression (12). Our goal is to address the need to build more empirically informed evidence at the population level about the specific ways that youth’s mental health is impacted by disasters. We provide further insight into the mental health effects of wildfires on youth by focusing on PTSD, depression, anxiety, alcohol/substance use, and resilience.

By analyzing school mental health survey data collected by Fort McMurray Public and Catholic School boards in November 2017 (18 months after the 2016 wildfire), we were able to help determine the overall population mental health effects in youth. This analysis allowed specific insights into measures of symptoms indicative of PTSD, depression, anxiety, alcohol and substance misuse, tobacco use, levels of self-esteem, quality of life, and resilience. In addition to capturing information on the mental health effects using standardized scales, the Fort McMurray schools were interested in understanding how other factors may influence these measurements. Therefore, in the survey, youth were also asked specific questions about their personal exposure to the wildfire and about the direct impact the wildfire had on their lives, including if they were present and witnessed the wildfire when it occurred, as well as if their own home was destroyed by the wildfire.

We published an initial analysis (22) of the November 2017 Fort McMurray mental health survey dataset, investigating depression, anxiety, alcohol/substance misuse, self-esteem, and quality of life and comparing the Fort McMurray data to a control (non-disaster) dataset collected from Red Deer, Alberta. This initial study found that symptoms of depression and anxiety were higher in the Fort McMurray sample than in the Red Deer sample [see Figure 2 in Ref. (22)]. Rates of probable depression were higher in Fort McMurray, though rates of probable anxiety and probable alcohol/substance use disorder were not significantly different. Self-esteem and quality of life scores were lower in the Fort McMurray group. The initial study (22) did not compare PTSD symptoms between Fort McMurray and Red Deer, as the Red Deer mental health survey did not include a PTSD measure.

The current study presents an extended analysis of the Fort McMurray dataset, including an investigation of PTSD as well as examining the effects of severity of impacts from the 2016 wildfire experienced by different individuals on PTSD, depression, anxiety, alcohol/substance misuse, self-esteem, quality of life, and resilience.

Based on previous research, our hypotheses were that more serious exposure to the wildfire and more direct impacts from the wildfire would have greater negative effects on youth’s mental health. More specifically, we hypothesized that these youth would have higher risk of having clinically significant scores on measures of PTSD, depression, anxiety, and alcohol/substance misuse, while having lower scores on measures of self-esteem, quality of life, and resilience.

## Materials and Methods

### Overview and Ethical Considerations

The two school boards in Fort McMurray—Fort McMurray Public Schools and Fort McMurray Catholic Schools—asked all students in Grade 7–Grade 12 to complete a comprehensive survey of their mental health, in November 2017, 18 months after the 2016 wildfire. The survey was conducted to enable the school boards to evaluate the effectiveness of the programs that they put in place immediately following the wildfire (for details, see Appendix—Mental Health Support Programs in the **Supplementary Materials**). The November 2017 survey time was determined by the schools based on logistical and staff capacity considerations. All aspects of survey data collection, including participant consent, were administered by Fort McMurray Public Schools and Fort McMurray Catholic Schools, in accordance with their standard procedures and policies. Researchers from the University of Alberta were asked to assist in designing the survey and analyzing the survey data. After the survey data were collected by the schools, researchers from the University of Alberta analyzed an anonymized version of the data. Survey data analysis was approved by the University of Alberta’s Health Research Ethics Board on June 26^th^, 2017 (ethics protocol number Pro00072669).

The survey included questions to determine demographics, mental health, resilience, and personal exposure to and direct impacts of the wildfire. All survey data were collected by the two Fort McMurray school systems, under their ethical guidelines and supervision. The survey was administered as part of the schools’ standard curriculum to assess the educational and support programs they established after the wildfire. The selection of measurement instruments was made by the school systems, informed by the existing literature in this context as well as expert advice from the University of Alberta research team. Parents and guardians were made aware of the survey by written letter 2 weeks prior to survey administration in the schools. Parents/guardians had the option to opt their child(ren) out of the survey if they desired. Students, themselves, had the option to participate or not in the survey, which was explained at the start of each survey data collection session (see details below as well as the survey description script in the **Supplementary Material**). Survey participation was anonymous, and participants were not asked for their names. This paper presents an analysis of the survey data collected from both school boards.

### Survey Questionnaires

The survey included the following 10 questionnaires (**Table 1**):

1. Demographics Questionnaire (Demographics, 7 questions) was a custom questionnaire assessing age, gender, the student’s grade, and school.
2. The Impact of Fire Questionnaire (IOF, 6 questions) was a custom questionnaire assessing the impact of the 2016 wildfire on the student, including whether they were present in Fort McMurray during the fire, whether they were evacuated, whether they personally saw the fire, and whether their home was destroyed.
3. Child PTSD Symptom Scale (CPSS, 19 questions) assesses symptoms of PTSD (23) and generates a score of PTSD symptom severity from 0 to 51.
4. The Patient Health Questionnaire, Adolescent version (PHQ-A, 11 questions) assesses symptoms of depression and suicidality (24, 25) and generates a score for depression symptom severity from 0 to 27.
5. The Hospital Anxiety and Depression Scale (HADS, 7 questions, anxiety-related questions only) assesses symptoms of anxiety (26) and generates a score for anxiety symptom severity from 0 to 21.
6. The CRAFFT Questionnaire (CRAFFT, 9 questions) assesses symptoms of alcohol and substance misuse (27, 28) and generates a score of alcohol/substance misuse severity from 0 to 6.
7. Tobacco Use Questionnaire (2 questions) includes two questions on tobacco use: “Over the past month: Do you smoke tobacco products? Do you use smokeless tobacco products?”
8. The Rosenberg Self-Esteem Scale (Rosenberg, 10 questions) assesses self-esteem (29) and generates a self-esteem score from 0 to 30.
9. The Kidscreen Questionnaire (Kidscreen-10, 11 questions) assesses quality of life (30) and generates a quality of life score from 0 to 44.
10. The Child and Youth Resilience Measure (CYRM-12, 12 questions) assesses resilience to adverse experience or trauma (31) and generates a resilience score from 12 to 60.

**Table 1:** Questionnaire details.

**Demographics Questionnaire**		
	Questions	Answer choices
1	Are you at school right now, while you are taking the survey?	Yes, no
2	Are you a student?	Yes, no
3	What gender do you identify with?	Female, male, other, prefer not to say
4	What is your age in years?	10 years or less, 11 years, 12 years, 13 years, 14 years, 15 years, 16 years, 17 years, 18 years, 19 years, 20 years or more
5	What is your school?	7, 8, 9, 10, 11, 12, other
6	What grade are you in?	Select from a list of all Ft McMurray schools with any classes in grades 7-12
7	What school were you in for grade 6?	Select from a list of all Ft McMurray schools with grade 6
**Impact of Fire Questionnaire**		
	Questions	Answer choices
1	Were in you or near Fort McMurray during any part of the 2016 wildfire?	Yes, no
2	Did you evacuate because of the fire?	Yes, no
3	Was your home destroyed by the fire?	Yes, no
4	Did you see the fire in person?	Yes, no
5	What school are you in?	Select from a list of all Ft McMurray schools with any classes in grades 7-12
6	What grade are you in?	7, 8, 9, 10, 11, 12, other
**Patient Health Questionnaire (PHQ-A, Depression Symptoms)**		
	Questions	Answer choices
	Over the past 2 weeks, how often have you been bothered by any of the following problems?	
1	Feeling down, depressed, irritable or hopeless	Not at all, Several days, More than half the days, Nearly every day
2	Little interest or pleasure in doing things?	Not at all, Several days, More than half the days, Nearly every day
3	Trouble falling or staying asleep, or sleeping too much	Not at all, Several days, More than half the days, Nearly every day
4	Poor appetite, weight loss, or overeating?	Not at all, Several days, More than half the days, Nearly every day
5	Feeling tired, or having little energy?	Not at all, Several days, More than half the days, Nearly every day
6	Feeling bad about yourself-or that you are a failure or that you have let yourself or your family down	Not at all, Several days, More than half the days, Nearly every day
7	Trouble concentrating on things, such as school work, reading or watching television	Not at all, Several days, More than half the days, Nearly every day
8	Moving or speaking so slowly that other people could have noticed. Or the opposite-being so figety or restless that you have been moving around a lot more than usual	Not at all, Several days, More than half the days, Nearly every day
9	Thoughts that you would be better off dead, or of hurting yourself in some way	Not at all, Several days, More than half the days, Nearly every day
	**Questions 10 and 11 asked only if answer to question 9 was "Several days", "More than half the days", or "Nearly everday"**	
10	Has there been a time in the past month when you have had serious thoughts about ending your life?	Yes, no
11	Have you ever, in your WHOLE LIFE, tried to kill yourself or made a suicide attempt?	Yes, no
**Hospital Anxiety and Depression Scale (HADS, Anxiety Symptoms)**		
	Questions	Answer choices
	Tick the box beside the reply that is closest to how you have been feeling in the past week. Don’t take too long over you replies: your immediate is best.	
1	I feel tense or wound up:	Most of the time; A lot of the time; From time to time, occasionally; Not at all
2	I get a sort of frightened feeling as if something bad is about to happen:	Very definitely and quite badly; Yes, but not too badly; A little, but it doesn’t worry me; Not at all
3	Worrying thoughts go through my mind:	A great deal of the time; A lot of the time; From time to time, but not too often; Only occasionally
4	I can sit at ease and feel relaxed:	Definitely; Usually; Not often; Not at all
5	I get a sort of frightened feeling like ‘butterflies’ in the stomach:	Not at all; Occasionally; Quite often; Very often
6	I feel restless and have to be on the move:	Very much indeed; Quite a lot; Not very much; Not at all
7	I get sudden feelings of panic:	Very often indeed; Quite often; Not very often; Not at all
**Child PTSD Symptom Scale (CPSS)**		
	Questions	Answer choices
	Instructions to participant: Below is a list of problems that kids sometimes have after experiencing an upsetting event. Read each one carefully and circle the number (0–3) that best describes how often that problem has bothered you IN THE LAST 2 WEEKS.	
1	Please select your most distressing event:	2016 Fort McMurray wildfire; Death of someone close to you; Injury that you suffered; Physical assault against you; Sexual assualt; Other
2	How long as it been since the event (in years)?	less than 1 month; 2-5 months; 6-11 months; 1 year; 2 years; 3-5 years; 6-10 years; 11 or more years
	Below is a list of problems that kids sometimes have after experiencing an upsetting event. Read each one carefully and circle the number (0–3) that best describes how often that problem has bothered you IN THE LAST 2 WEEKS.	
3	Having upsetting thoughts or images about the event that came into your head when you didn’t want them to	Not at all or only at one time; Once a week or less/ once in a while; 2 to 4 times a week/ half the time; 5 or more times a week/almost always
4	Having bad dreams or nightmares	Same as above
5	Acting or feeling as if the event was happening again (hearing something or seeing a picture about it and feeling as if I am there again)	Same as above
6	Feeling upset when you think about it or hear about the event (for example, feeling scared, angry, sad, guilty, etc)	Same as above
7	Having feelings in your body when you think about or hear about the event (for example, breaking out into a sweat, heart beating fast)	Same as above
8	Trying not to think about, talk about, or have feelings about the event	Same as above
9	Trying to avoid activities, people, or places that remind you of the traumatic event	Same as above
10	Not being able to remember an important part of the upsetting event	Same as above
11	Having much less interest or doing things you used to do	Same as above
12	Not feeling close to people around you	Same as above
13	Not being able to have strong feelings (for example, being unable to cry or unable to feel happy)	Same as above
14	Feeling as if your future plans or hopes will not come true (for example, you will not have a job or getting married or having kids)	Same as above
15	Having trouble falling or staying asleep	Same as above
16	Feeling irritable or having fits of anger	Same as above
17	Having trouble concentrating (for example, losing track of a story on the television, forgetting what you read, not paying attention in class)	Same as above
18	Being overly careful (for example, checking to see who is around you and what is around you)	Same as above
19	Being jumpy or easily startled (for example, when someone walks up behind you)	Same as above
**CRAFFT Questionnaire (Drugs/Alcohol/Tabacco)**		
	Questions	Answer choices
	During the past 12 months, did you:	
1	Drink any alcohol (more than a few sips)?	Yes, no
2	Smoke any marijuana or hashish?	Yes, no
3	Use anything else to get high?	Yes, no
4	Have you ever ridden in a CAR driven by someone (including yourself) who was “high” or had been using alcohol or drugs?	Yes, no
	**Questions 5-9 asked only if “yes” to one or more of questions 1-3.**	
5	Do you ever use alcohol or drugs to RELAX, feel better about yourself, or fit in?	Yes, no
6	Do you ever use alcohol or drugs while you are by yourself, or ALONE?	Yes, no
7	Do you every FORGET things you did while using alcohol or drugs?	Yes, no
8	Do your FAMILY or FRIENDS ever tell you that you should cut down on your drinking or drug use?	Yes, no
9	Have you ever gotten into TROUBLE while you were using alcohol or drugs?	Yes, no
**Tobacco Use Questionnaire**		
	Questions	Answer choices
	During the past month:	
1	Do you smoke tobacco products?	Yes, no
2	Do you use smokeless tobacco products?	Yes, no
**Rosenberg Self-Esteem Scale**		
	Questions	Answer choices
1	On the whole, I am satisfied with myself.	Strongly agree, Agree, Disagree, Strongly disagree
2	At times, I think I am no good at all.	Strongly agree, Agree, Disagree, Strongly disagree
3	I feel that I have a number of good qualities.	Strongly agree, Agree, Disagree, Strongly disagree
4	I am able to do things as well as most other people.	Strongly agree, Agree, Disagree, Strongly disagree
5	I feel I do not have much to be proud of.	Strongly agree, Agree, Disagree, Strongly disagree
6	I certainly feel useless at times.	Strongly agree, Agree, Disagree, Strongly disagree
7	I feel that I’m a person of worth, at least on an equal plane with others.	Strongly agree, Agree, Disagree, Strongly disagree
8	I wish I could have more respect for myself.	Strongly agree, Agree, Disagree, Strongly disagree
9	All in all, I am inclined to feel that I am a failure.	Strongly agree, Agree, Disagree, Strongly disagree
10	I take a positive attitude toward myself.	Strongly agree, Agree, Disagree, Strongly disagree
**Kidscreen Questionnaire (Quality of Life)**		
	Questions	Answer choices
	Thinking about the last week:	
1	Have you physically felt fit and well?	Not at all, slightly, moderately, very, extremely
2	Have you felt full of energy?	Never, seldom, quite often, very often, always
3	Have you felt sad?	Never, seldom, quite often, very often, always
4	Have you felt lonely?	Never, seldom, quite often, very often, always
5	Have you had enough time for yourself?	Never, seldom, quite often, very often, always
6	Have you been able to do the things that you want to do in your free time?	Never, seldom, quite often, very often, always
7	Have your parent(s) treated you fairly?	Never, seldom, quite often, very often, always
8	Have you had fun with your friends?	Never, seldom, quite often, very often, always
9	Have you got on well at school?	Not at all, slightly, moderately, very, extremely
10	Have you been able to pay attention?	Never, seldom, quite often, very often, always
11	In general, how would you say your health is?	Excellent, very good, good, fair, poor
**Child and Youth Resilience Measure (CYRM-12)**		
	Questions	Answer choices
	To what extent do the sentences below describe you? Select an answer for each statement.	
1	I am able to solve my problems without harming myself or others	Not at all; A little; Some-what; Quite a bit; A lot
2	I know where to go in the community to get help	Not at all; A little; Some-what; Quite a bit; A lot
3	Getting an education is important to me	Not at all; A little; Some-what; Quite a bit; A lot
4	I try to finish what I start	Not at all; A little; Some-what; Quite a bit; A lot
5	I have people I look up to	Not at all; A little; Some-what; Quite a bit; A lot
6	My parents/caregivers know a lot about me	Not at all; A little; Some-what; Quite a bit; A lot
7	My family stands by me during difficult times	Not at all; A little; Some-what; Quite a bit; A lot
8	My friends stand by me during difficult times	Not at all; A little; Some-what; Quite a bit; A lot
9	I have opportunities to develop skills that will be useful later in life	Not at all; A little; Some-what; Quite a bit; A lot
10	I am treated fairly in my community	Not at all; A little; Some-what; Quite a bit; A lot
11	I feel I belong at school	Not at all; A little; Some-what; Quite a bit; A lot
12	I enjoy my cultural and family traditions	Not at all; A little; Some-what; Quite a bit; A lot

### Survey Administration Procedure

Students participated in the survey during regular school hours in the vast majority of cases. (A few students with special circumstances participated from home, using their own computers.) Each participant used a laptop or desktop computer to fill in the survey. The survey website used an HTML/CSS front-end and a back-end server written in the Clojure programming language (http://clojure.org). (Clojure was chosen as the server language for its high productivity with small development teams and strong track record in web applications.) Students either came to a computer laboratory or used laptops brought to their classroom, depending on their school. A survey description script was read to each class at the beginning of each survey session (reproduced in the **Supplementary Material**). The script explained the purpose of the survey, how to complete the survey, participant confidentiality, anonymity, and voluntary participation. Students’ confidential, anonymous, and voluntary participation in the survey was emphasized. Before participating, students were also given the opportunity to ask questions. Survey participation was anonymous, and the survey did not ask participants for their names. The survey battery included a total of 96 questions. Participation required less than 20 min for most students, though a small number of students took up to 50 min. Participants were able to skip questions, although the survey description script encouraged them to answer all questions.

### Cutoff Scores and Probable Diagnoses

For each participant, we derived probable diagnoses of four different psychiatric conditions from the participant’s questionnaire answers on specific scales, combined with previously established cutoff points for probable diagnoses appropriate to each scale. Specifically, we considered PTSD (from the CPSS scale), depression (from PHQ-A), anxiety (from HADS), and alcohol/substance use disorder (from CRAFFT). We use the term “probable diagnosis” because scores were based on self-report scales, not psychiatric clinical interviews, and scores on a specific scale are not clinically diagnostic. Nonetheless, existing literature reports good correspondence between psychiatric clinical diagnoses of PTSD, depression, anxiety, and alcohol/substance use disorder with probable diagnoses based on widely published cutoff scores for the above four scales (27, 28, 32–35). Each of the four probable diagnoses was defined based on a threshold value for the appropriate scale. Thus, probable PTSD was defined as having a CPSS score of 15 or more (35, 36). Probable depression was defined as having a PHQ-A score of 11 or more (33). Probable moderately severe depression was defined as having a PHQ-A score of 15 or more (32). Suicidal thinking was assessed from responses to questions 9 and 10 from the PHQ-A: question 9 “Over the past 2 weeks, how often have you been bothered by any of the following problems: Thoughts that you would be better off dead, or of hurting yourself in some way?” and question 10 “Has there been a time in the past month when you have had serious thoughts about ending your life?” Participants were defined as positive for suicidal thinking if they answered “Several days,” “More than half the days,” or “Nearly every day” to PHQ-A question 9 and “Yes” to question 10. Participants who answered “Not at all” to question 9 were not given question 10 and were defined as negative for suicidal thinking. Participants who answered “Several days,” “More than half the days,” or “Nearly every day” to PHQ-A question 9 and “No” to question 10 were defined as negative for suicidal thinking (as distinct from thinking about self-harm). (PHQ-A question 11 was not considered in the definition of suicidal thinking.) Probable anxiety was defined as having a HADS score of 11 or more (34). Probable alcohol/substance use disorder was defined as having a CRAFFT score of 2 or more (27, 28). Tobacco use was defined as answering “yes” to either of the two questions on the Tobacco Use Questionnaire. We also defined an “Any of 4 probable diagnoses” criterion as being positive for one or more of the four probable diagnoses: PTSD, depression, anxiety, or alcohol/substance use disorder.

### Statistical Analysis

We defined five pairs of subgroups from participants, namely: 1) no impact of fire vs. any impact of fire; 2) present during the fire vs. not present; 3) saw the fire in person vs. did not see it; 4) home destroyed by the fire vs. home not destroyed; and 5) high resilience vs. low resilience.

1. No impact of fire vs. any impact of fire: The no impact of fire group was defined as those participants answering “no” to the following four questions: “Were you in or near Fort McMurray during any part of the 2016 wildfire?,” “Did you evacuate because of the fire?,” “Did you see the fire in person?,” “Was your home destroyed by the fire?” The any impact of fire group was defined as those participants who answered “yes” to any one or more of those four questions. Participants who did not provide an answer to all four questions were excluded from both the no impact of fire group and the any impact of fire group.
2. Present during the fire vs. not present: The present and not present during the fire groups were defined as participants answering “yes” and “no,” respectively, to the question “Were you in or near Fort McMurray during any part of the 2016 wildfire?” Participants who did not answer that question were excluded from both groups.
3. Personally witnessed the fire vs. did not see it: The personally witnessed the fire vs. did not personally witness the fire were defined as participants answering “yes” and “no,” respectively, to the question “Did you see the fire in person?” Participants who did not answer that question were excluded from both groups.
4. Home destroyed by the fire vs. home not destroyed: The home destroyed vs. not destroyed by the fire groups were defined as participants answering “yes” and “no,” respectively, to the question “Was your home destroyed by the fire?” Participants who did not answer that question were excluded from both groups.
5. High resilience vs. low resilience: Resilience was measured with the CYRM-12 questionnaire. High and low resilience groups were defined as participants whose CYRM-12 scores were, respectively, equal/above the median or below the median CYRM-12 score.

For each of these five pairs of groups, we compared the following 15 measures: 1) mean CPSS score, 2) mean PHQ-A score, 3) mean HADS score, 4) mean CRAFFT score, 5) mean Rosenberg score, 6) mean Kidscreen score, 7) mean CYRM-12 score, 8) percent probable PTSD, 9) percent probable depression, 10) percent probable moderately severe depression, 11) percent suicidal thinking, 12) percent probable anxiety, 13) percent probable alcohol/substance use disorder, 14) percent tobacco use, and 15) percent any of four probable diagnoses. Details of groups are given above. Details of questionnaire scores and probable diagnoses are given above. (Note that we did not compare mean CYRM-12 resilience scores for high vs. low resilience participants, as this would have been tautological.) In analyzing data for a given measure (e.g., mean CPSS), we included only those participants who provided answers for all questions in the relevant questionnaire or scale.

Permutation testing was used for all statistical comparisons (#iterations = 10^5^). Permutation testing is a nonparametric method, chosen for its robustness against non-normality. All tests were two-sided, two-sample tests, with a null hypothesis of no difference between the means of the two groups for the given comparison. In total, our analysis included 74 individual statistical tests. We addressed multiple comparisons using the Benjamini–Hochberg method for false discovery rate (FDR) correction. This method computed a threshold of p = 0.027 for FDR correction. Effect sizes reported in tables are Cohen’s d (mean difference divided by pooled standard deviation). We performed all analyses using in-house computer code in the Clojure programming language (http://clojure.org). The server for the questionnaire website was written in Clojure, and the server saved participants’ (anonymous, encrypted) questionnaire answers in Clojure data structures. It was therefore simplest to analyze the data in Clojure as well. In-house analysis code included data collating, sorting, filtering, and questionnaire scoring functions as well as the permutation testing and Benjamini–Hochberg FDR algorithms, which are straightforward to implement. The code for statistical testing and FDR correction is available at http://github.com/mbrown/mrgbstats.

There were small, not statistically significant differences in the distributions of gender identification in the pairs of groups (see **Supplementary Table 1** in the **Supplementary Material**). To test whether the differences in gender identification, though not significant, may have influenced the statistical comparisons of interest, we re-ran all of the comparisons on subsets of the data that were subsampled so as to make the distributions of gender identification as similar as possible between the groups. The gender-based subsampling did not qualitatively change the significant results; statistical comparisons that were significant in the original dataset were also significant in the subsampled dataset. In addition, there were small but, in some cases, statistically significant differences in mean age in the pairs of groups (see **Supplementary Table 1** in the **Supplementary Material** for full details). To test for confounding effects of age, we re-ran all of the statistical comparisons on subsets of the data that were subsampled to make the distributions of ages as similar as possible between the groups (including no significant difference in mean age). The age-based subsampling did not qualitatively alter the significant results.

## Results

The survey was administered to the entire available population of Grades 7–12 students in Fort McMurray, Alberta, Canada who were attending either the public schools (48% of students) or Catholic schools (52% of students) and who were attending their schools on the day the survey was conducted. Five public schools and two Catholics schools took part in the survey. In total, 3,252 students participated out of 4,407 students enrolled in both the public and Catholic systems in Grades 7–12. That is, a total of 72% of enrolled students participated in the survey. The results presented below are organized by comparison groups. For convenience, **Supplementary Table 2** shows the results organized by mental health condition.

### Data Exclusion

Of the 3,252 students who participated in the survey, data from 182 students were excluded due to one or more of the following exclusion criteria:

1. Age less than or equal to 10 years.
2. Age greater than or equal to 20 years.
3. Inconsistent answers among the positive and negative questions from the Rosenberg questionnaire (see details in **Supplementary Material**).
4. Inconsistent answers among the positive questions from the Rosenberg questionnaire and the positive questions from the Kidscreen questionnaire (see details in **Supplementary Material**).
5. Inconsistent answers among the nonreversed and reversed questions from the HADS questionnaire. (Answer order for two of the HADS questions is reversed to test for consistency; see details in **Supplementary Material**.)

Criteria 3 to 5 above excluded participants who gave inconsistent answers, possibly because they were not paying attention to the survey or did not understand the questions. After exclusions, the dataset included 3,070 participants, and statistical analysis was done on this set of participant data.

### Demographics

Demographics for the 3,070 participants were as follows. Gender identification was 48% female, 48% male, 2% other, and 2% preferred not to say. Age ranged from 11 to 19; mean age was 14.32 with standard deviation 1.82.

### Overall Scores and Rates of Probable Diagnoses

**Table 2** shows mean questionnaire scores as well as rates for each probable diagnosis category across the entire set of 3,070 participants. For each questionnaire or probable diagnosis, only those participants who filled in every question of the relevant questionnaire were included (see N column in **Table 2**).

**Table 2:** Overall Questionnaire Scores and Rates of Probable Diagnoses.

Measure	N	Score
CPSS score (PTSD)	2877	12.8 +/- 11.5
PHQ-A score (depression)	2970	8.0 +/- 6.5
HADS score (anxiety)	2990	7.7 +/- 4.7
CRAFFT score (alcohol/drugs)	3001	0.55 +/- 1.25
Rosenberg score (self-esteem)	2974	18.2 +/- 6.6
Kidscreen score (quality of life)	2984	27.0 +/- 8.2
CYRM-12 score (resilience)	2937	46.5 +/- 9.2
Measure	N	Rate
Probable PTSD	2877	37%
Probable depression	2970	31%
Probable moderately severe depression	2970	17%
Suicidal thinking	2998	16%
Probable anxiety	2990	27%
Probable alcohol/substance use disorder	3001	15%
Tobacco use	3011	13%
Any of 4 probable diagnoses	3070	46%

N = number subjects who filled in all questions in relevant questionnaire.

Scores are means +/- standard deviation (not standard error).

Overall, the survey findings reveal the following: PTSD scores were available from 2,877 students, and 37% met criteria for probable PTSD (CPSS score ≥ 15). There were 2,970 students for whom depression scores were available, and of these, 31% met criteria for probable depression (PHQ-A score ≥ 11), and 17% met criteria for probable depression of at least moderate severity (PHQ-A score ≥ 15). Of these 2,970 students, 16% exhibited suicidal thinking. Anxiety scores were available for 2,990 students, of whom 27% met criteria for probable anxiety (HADS score ≥ 11). Alcohol/substance use scores were available from 3,001 students, of whom 15% met criteria for probable alcohol/substance use (CRAFFT score ≥ 2). Tobacco use data were available from 3,011 students, and 13% exhibited tobacco use. Of the 3,070 students, 46% satisfied the “any of 4 probable diagnoses” criterion. (That is, all 3,070 students filled out at least one of the diagnostic questionnaires, and 46% met criteria for one or more probable diagnosis of PTSD, depression, anxiety, or alcohol/substance use).

### No Impact of Fire Vs. Any Impact of Fire

There were no statistically significant differences between the “no impact of fire” group and the “any impact of fire” group in 14 of the 15 measures compared (**Table 3**). CYRM-12 resilience scores were slightly higher for the “any impact of fire” group (p = 0.0041, see **Table 3**). Suicidal thinking was higher for the “any impact of fire” group (p = 0.036), but this result did not survive FDR correction for multiple comparisons. Each comparison used only those students who filled out all questions in the relevant questionnaire. Numbers of students ranged from 258 to 275 for the “no impact of fire” category and 2,610 to 2,705 for the “any impact of fire” category.

**Table 3:** No impact of fire vs. any impact.

Measure	No impact of fire	Any impact of fire	P-value	Effect size
	Score	Score		
CPSS score (PTSD)	13.4 +/- 11.6	12.8 +/- 11.5	0.39	0.06
PHQ-A score (depression)	8.0 +/- 6.1	8.0 +/- 6.5	0.89	-0.01
HADS score (anxiety)	7.7 +/- 4.7	7.7 +/- 4.7	0.95	0.00
CRAFFT score (alcohol/drugs)	0.56 +/- 1.32	0.55 +/- 1.24	0.92	0.01
Rosenberg score (self-esteem)	17.8 +/- 6.0	18.3 +/- 6.6	0.31	-0.06
Kidscreen score (quality of life)	26.4 +/- 7.6	27.0 +/- 8.3	0.23	-0.08
CYRM-12 score (resilience)	44.9 +/- 9.7	46.6 +/- 9.1	0.0042*	-0.19
Measure	Rate	Rate	P-value	
Probable PTSD	39%	37%	0.50	
Probable depression	32%	31%	0.58	
Probable moderately severe depression	15%	17%	0.35	
Suicidal thinking	11%	16%	0.036	
Probable anxiety	26%	27%	0.72	
Probable alcohol/substance use disorder	14%	15%	0.79	
Tobacco use	13%	12%	0.77	
Any of 4 probable diagnoses	49%	47%	0.44	

*p-value survives FDR multiple comparison correction (threshold 0.027).

Scores are means +/- standard deviation (not standard error).

### Present During the Fire Vs. Not Present

There were no statistically significant differences between the “present in Fort McMurray during the fire” group vs. the “not present” group in 13 of the 15 measures compared (**Table 4**). CYRM-12 resilience scores were slightly higher for the “present during the fire” group (p = 0.0025, see **Table 4**). Rates of suicidal thinking were higher for the “present during the fire” group (p = 0.026). Each comparison used only those students who filled out all questions in the relevant questionnaire. Numbers of students ranged from 2,570 to 2,663 for the “present in Fort McMurray during the fire” category and 305 to 334 for the “any impact of fire” category.

**Table 4:** Present vs. not present in Fort McMurray during fire.

Measure	Present during fire	Not present	P-value	Effect size
	Score	Score		
CPSS score (PTSD)	12.8 +/- 11.5	13.0 +/- 11.4	0.79	-0.02
PHQ-A score (depression)	8.1 +/- 6.5	7.8 +/- 6.1	0.51	0.04
HADS score (anxiety)	7.7 +/- 4.7	7.7 +/- 4.6	0.93	0.01
CRAFFT score (alcohol/drugs)	0.55 +/- 1.24	0.52 +/- 1.29	0.62	0.03
Rosenberg score (self-esteem)	18.2 +/- 6.6	18.1 +/- 5.9	0.74	0.02
Kidscreen score (quality of life)	27.0 +/- 8.3	26.5 +/- 7.7	0.27	0.06
CYRM-12 score (resilience)	46.7 +/- 9.1	45.0 +/- 9.7	0.0025*	0.18
Measure	Rate	Rate	P-value	
Probable PTSD	37%	37%	0.95	
Probable depression	31%	30%	0.90	
Probable moderately severe depression	17%	14%	0.21	
Suicidal thinking	16%	11%	0.026*	
Probable anxiety	27%	25%	0.47	
Probable alcohol/substance use disorder	15%	13%	0.32	
Tobacco use	13%	12%	0.79	
Any of 4 probable diagnoses	47%	47%	0.91	

*p-value survives FDR multiple comparison correction (threshold 0.027).

Scores are means +/- standard deviation (not standard error).

### Personally Witnessed the Fire vs. Did Not See It

Comparison of participants who personally witnessed the fire vs. those who did not see the fire revealed significant differences in 13 of the 15 measures tested (see **Table 5**). Participants who personally saw the fire exhibited higher mean scores for all mental health conditions tested, higher rates for all probable diagnoses tested, and lower means scores for quality of life. Rosenberg self-esteem and CYRM-12 resilience scores were not significantly different between these two groups. Each comparison used only those students who filled out all questions in the relevant questionnaire. Numbers of students ranged from 2,270 to 2,350 for the “personally witnessed fire” category and 604 to 641 for the “did not see the fire” category.

**Table 5:** Personally witnessed fire vs. did not see fire.

Measure	Witnessed fire	Did not see fire	P-value	Effect size
	Score	Score		
CPSS Score (PTSD)	13.2 +/- 11.6	11.6 +/- 11.0	0.0021*	0.14
PHQ-A Score (depression)	8.2 +/- 6.6	7.2 +/- 6.0	0.00046*	0.16
HADS Score (anxiety)	7.9 +/- 4.8	7.2 +/- 4.5	0.0023*	0.14
CRAFFT Score (alcohol/drugs)	0.60 +/- 1.29	0.35 +/- 1.06	0.00001*	0.20
Rosenberg Score (self-esteem)	18.1 +/- 6.7	18.6 +/- 6.1	0.076	-0.08
Kidscreen Score (quality of life)	26.8 +/- 8.3	27.7 +/- 7.8	0.019*	-0.11
CYRM-12 Score (resilience)	46.6 +/- 9.1	46.0 +/- 9.5	0.19	0.06
Measure	Rate	Rate	P-value	
Probable PTSD	38%	32%	0.0042*	
Probable depression	32%	26%	0.0075*	
Probable moderately severe depression	18%	13%	0.0032*	
Suicidal thinking	17%	12%	0.0011*	
Probable anxiety	28%	22%	0.0018*	
Probable alcohol/substance use disorder	16%	9%	0.00001*	
Tobacco use	14%	7%	0.00001*	
Any of 4 probable diagnoses	49%	41%	0.00022*	

*p-value survives FDR multiple comparison correction (threshold 0.027).

Scores are means +/- standard deviation (not standard error).

### Home Destroyed by the Fire Vs. Home Not Destroyed

Participants whose homes were destroyed vs. not destroyed by the fire exhibited significant differences in 12 of the 15 measures tested (see **Table 6**). Those whose home was destroyed had higher mean scores for all mental health conditions tested, higher rates for all probable diagnoses tested (except suicidal thinking), and lower means scores for self-esteem, quality of life, and resilience. Rates of probable depression and tobacco use were higher for the home destroyed group (p = 0.039 and p = 0.033, respectively), but these results did not survive FDR correction for multiple comparisons. Each comparison used only those students who filled out all questions in the relevant questionnaire. Numbers of students ranged from 284 to 299 for the “home destroyed” category and 2,590 to 2,691 for the “home not destroyed” category.

**Table 6:** Home destroyed vs. not destroyed by fire.

Measure	Home destroyed	Home not destroyed	P-value	Effect size
	Score	Score		
CPSS Score (PTSD)	15.9 +/- 12.5	12.5 +/- 11.3	0.00001*	0.30
PHQ-A Score (depression)	9.1 +/- 6.8	7.9 +/- 6.4	0.0018*	0.19
HADS Score (anxiety)	8.6 +/- 5.1	7.7 +/- 4.7	0.00083*	0.20
CRAFFT Score (alcohol/drugs)	0.78 +/- 1.44	0.52 +/- 1.22	0.0011*	0.20
Rosenberg Score (self-esteem)	17.2 +/- 6.8	18.3 +/- 6.5	0.0049*	-0.17
Kidscreen Score (quality of life)	25.4 +/- 8.7	27.2 +/- 8.1	0.00058*	-0.21
CYRM-12 Score (resilience)	44.8 +/- 10.0	46.7 +/- 9.1	0.0013*	-0.20
Measure	Rate	Rate	P-value	
Probable PTSD	46%	36%	0.00033*	
Probable depression	36%	30%	0.039	
Probable moderately severe depression	22%	16%	0.027*	
Suicidal thinking	19%	15%	0.11	
Probable anxiety	35%	26%	0.0023*	
Probable alcohol/substance use disorder	22%	14%	0.00019*	
Tobacco use	16%	12%	0.033	
Any of 4 probable diagnoses	58%	46%	0.00008*	

*p-value survives FDR multiple comparison correction (threshold 0.027).

Scores are means +/- standard deviation (not standard error).

### High Resilience Vs. Low Resilience

The median resilience score (CYRM-12 score) across the 3,070 participants included in the statistical analysis was 48. High resilience was defined as having a CYRM-12 score of 48 or more, while low resilience was defined as having a CYRM-12 score of less than 48. There were significant differences between participants with high vs. low resilience scores on all 15 measures tested (see **Table 7**). Participants with high resilience showed lower scores on all mental health conditions, lower rates for all probable diagnoses, and higher self-esteem and quality of life scores. Each comparison used only those students who filled out all questions in the relevant questionnaire. Numbers of students ranged from 1,480 to 1,513 for the high resilience category and 1,376 to 1,424 for the low resilience category.

**Table 7:** High vs. low resilience.

Measure	High resilience	Low resilience	P-value	Effect size
	Score	Score		
CPSS Score (PTSD)	7.9 +/- 8.1	18.1 +/- 12.2	0.00001*	-0.99
PHQ-A Score (depression)	5.2 +/- 4.6	11.0 +/- 6.8	0.00001*	-1.00
HADS Score (anxiety)	6.1 +/- 4.0	9.5 +/- 4.8	0.00001*	-0.79
CRAFFT Score (alcohol/drugs)	0.30 +/- 0.89	0.81 +/- 1.50	0.00001*	-0.42
Rosenberg Score (self-esteem)	21.6 +/- 5.2	14.6 +/- 6.0	0.00001*	1.24
Kidscreen Score (quality of life)	31.3 +/- 6.5	22.4 +/- 7.3	0.00001*	1.30
Measure	Rate	Rate	P-value	
Probable PTSD	18%	56%	0.00001*	
Probable depression	14%	49%	0.00001*	
Probable moderately severe depression	5%	30%	0.00001*	
Suicidal thinking	5%	27%	0.00001*	
Probable anxiety	14%	41%	0.00001*	
Probable alcohol/substance use disorder	8%	22%	0.00001*	
Tobacco use	8%	17%	0.00001*	
Any of 4 probable diagnoses	29%	67%	0.00001*	

*p-value survives FDR multiple comparison correction (threshold 0.027).

Scores are means +/- standard deviation (not standard error).

## Discussion

### Mental Health

This study examined the impact of the wildfire on the mental health of youth in Fort McMurray, Alberta. The findings indicate rates of probable PTSD (37%), probable depression (31%), probable anxiety (27%), and probable alcohol/substance use disorder (15%) in the population of Grades 7–12 students in Fort McMurray, Alberta 18 months after the 2016 wildfire. By way of comparison with previous Canadian studies, the prevalence of probable diagnosis of PTSD in children has been reported as 15.5% (35); prevalence of major depression in adolescents has been reported as 4–8% (37); incidence of probable anxiety in children and adolescents has been reported as 10–13% (38); and 17% of adolescents have reported binge drinking in the past month (39). In addition, comparison of the Fort McMurray survey data with control (nondisaster) data from Red Deer, Alberta (22) found that symptoms of depression and anxiety were higher in the Fort McMurray data as were rates of probable depression [see Figure 2 in Ref. (22)]. Rates of probable anxiety and probable alcohol/substance use disorder were not significantly different (22). Self-esteem and quality of life scores were lower in Fort McMurray (22). [The earlier study (22) did not compare PTSD symptoms between Fort McMurray and Red Deer, as the Red Deer mental health survey did not include a PTSD measure.] These findings therefore suggest significant elevated rates of PTSD, probable depression, and anxiety in Fort McMurray students following the wildfire. Overall, the results presented in the current study are consistent with previous studies showing increased mental health symptoms subsequent to wildfires (4–8, 11, 13, 40). These results highlight the need for mental health programs and supports for youth following disaster. (Consistent with this, Fort McMurray Public and Catholic Schools have put significant and ongoing work into establishing and maintaining mental health support programs for their students in the period following the wildfire. For details, see Appendix—Mental Health Support Programs in the **Supplementary Materials**).

### Specific Impacts of the Wildfire

It was anticipated that those students who had the greatest level of impact from the 2016 wildfire, in the form of personally seeing the fire (vs. not seeing it) or having their homes destroyed (vs. not destroyed), would have more frequent mental health symptomatology. The findings indicate that students directly impacted by the fire had significantly higher scores on scales measuring symptoms related to PTSD, depression, anxiety, and substance misuse. These students also had significantly lower scores for self-esteem, quality of life, and resilience. The findings indicate that students directly impacted by the fire had higher rates of probable PTSD, depression, anxiety, and substance use disorder and higher rates of suicidal thinking and tobacco use. These results suggest that mental health impacts are more severe with increased severity of impact from wildfire on the individual, in the form of personally seeing the fire or having one’s home destroyed.

Importantly, the findings also revealed an unexpected pattern in students who had no direct impact from the fire; that is, they were out of town during the fire for a variety of reasons or were not living in Fort McMurray during the fire. The scores on almost all scales and questionnaires for these students were very similar to those who had direct experience with any impact of the fire (present during the fire, saw the fire, and/or home destroyed) in terms of mental health symptom scores, self-esteem, quality of life scores, and rates of probable diagnoses. These findings suggest that youth not directly impacted by the fire nonetheless experienced vicarious trauma as a result of the fire’s large impact on the community, similar to secondary trauma experienced by first responders and researchers in post-disaster situations (41, 42). These results are consistent with the literature, which indicates a substantial deleterious impact of wildfire disasters on population mental health (4–6, 8–11, 40). These results emphasize the need for policies and programs to support mental health in youth who are directly or indirectly impacted by disaster, as well as the need for a community-wide approach.

### Resilience

Finally, resilience also played an important role in the mental health of youth. Low resilience was linked with substantially more severe mental health impacts from the wildfire. Though we do not have survey data from Fort McMurray prior to the 2016 wildfire, we believe that having a higher baseline level of individual resilience prior to a disaster would have beneficial effects on youth’s mental health. This is supported by previous research showing that higher resilience is associated with better mental health outcomes following disaster (9, 13, 20, 21). These results emphasize the need for long-term mental health supports for youth post-disaster, with specific efforts focusing on increasing youth’s resilience, which may serve as a protective factor that mitigates the effects of disaster on mental health.

### Implications

In terms of interventions, there is often active discussion about whether to intervene in complete populations (so-called Universal interventions), even when only a subset of the population has issues, or whether is it better to be more focused (so-called Targeted or Selective interventions) for those with the greatest need. The issue with the latter approach, however, is that it misses large numbers of individuals who have only mild or moderate symptoms. The approach we have previously advocated is a combined approach, and we have shown this to be successful (43, 44). Nonetheless, we are not aware that this comprehensive approach has been examined after a wildfire or other major event, so its efficacy in this situation remains uncertain.

### Limitations

The results presented here are based on a large sample of 3,070 participants. One limitation of the study is that clinical measures were based on self-report questionnaires as opposed to clinical interviews. Conducting a full clinical interview with each participant would not have been feasible given the large sample size. It would have been useful to have data using the same questionnaire battery from the Fort McMurray student population from before the 2016 Wildfire. This would have allowed a before and after wildfire comparison with identical instruments. Instead, we have previously presented a comparison of the Fort McMurray mental health data with control data previously collected using a very similar battery in Red Deer, Alberta (22).

## Conclusion

In conclusion, the present results support existing findings that both youth and communities impacted by a wildfire, or similar major disaster, experience long-term mental health impacts. The present data extend this by examining all youth in grades 7–12 attending schools in Fort McMurray following the 2016 wildfire and identifying those groups who are most vulnerable and have the greatest risk. It is likely that providing additional assistance for all individuals to increase resilience, and also focusing on those with particularly severe wildfire impact, would be useful in terms of improving population mental health after a wildfire and other major disasters. Fort McMurray Public and Catholic Schools have put significant and ongoing work into establishing and maintaining mental health support programs for students in the aftermath of the 2016 wildfire. The findings presented here are consistent with the need for long-term mental health supports in disaster-affected communities such as Fort McMurray.

## Data Availability

The dataset analyzed in this study is available from the corresponding author on reasonable request.

## Ethics Statement

In November 2017, 18 months after the 2016 wildfire, the two school boards in Fort McMurray—Fort McMurray Public Schools and Fort McMurray Catholic Schools—asked all students in Grade 7–Grade 12 to complete a comprehensive survey of their mental health. The survey was conducted to enable the school boards to evaluate the effectiveness of the programs they had put in place immediately following the wildfire. Researchers from the University of Alberta were asked to collaborate and provide assistance in designing the survey and analyzing the survey data. After the data were collected, the anonymized data were made available for analysis by the researchers from the University of Alberta. The analysis of the survey data was approved by the University of Alberta’s Health Research Ethics Board on June 26th, 2017 (ethics protocol number Pro00072669).

The survey included questions to determine demographics, mental health, resilience, personal exposure to, and direct impacts of the wildfire. All survey data were collected under the auspices and ethical guidelines of the two Fort McMurray school systems and were administered as part of their standard curriculum and as an ongoing assessment of the educational and support programs they had put in place after the wildfire. The selection of measurement instruments was determined by the school systems and was informed by the existing scholarly literature and findings. Parents and guardians were notified of the process by written letter 2 weeks prior to the administration of the survey in the schools, and they had the option to opt their child(ren) out of the survey. Students had the option to participate or not in the survey, and this was explained at the start of each survey data collection session. Survey participation was anonymous; participants were not asked for their names. This paper provides an analysis of the survey data that were collected from both school boards.

## Author’s Note

Some portions of the manuscript, mostly in the *Materials and Methods* section, are shared with a separate manuscript by the authors (22) presenting a distinct set of findings based on a separate analysis comparing the data from Fort McMurray to a similar survey dataset collected previously in Red Deer, Alberta.

## Author Contributions

MB, VA, AG, PB-M, JD, CM-H, JO, MM, SN, DK, and PS were in charge of study design. MM, SN, and DK were in charge of data collection. Analysis was done by MB, IC, and PS. Manuscript preparation was done by MB, IC, PB-M, JD, CM-H, and PS.

## Funding

Funding for support to the Fort McMurray schools in developing their survey and support for statistical analysis was provided by collaborative grant funding from the Canadian Institutes of Health Research, Canadian Red Cross, and Alberta Innovates Health Solutions (grant number 201600546).

## Conflict of Interest Statement

The authors declare that the research was conducted in the absence of any commercial or financial relationships that could be construed as a potential conflict of interest.

## Abbreviations

CPSS, Child PTSD Symptom Scale; CRAFFT, CRAFFT Questionnaire (proper name of the questionnaire is CRAFFT); CYRM-12, Child and Youth Resilience Measure; EMPATHY, Empowering a Multimodal Pathway Towards Healthy Youth project; FDR, false discovery rate; HADS, Hospital Anxiety and Depression Scale; Kidscreen-10, Kidscreen Questionnaire; PHQ-A, The Patient Health Questionnaire, Adolescent version; PTSD, post-traumatic stress disorder; Rosenberg, Rosenberg Self-Esteem Scale.

## References

[1] CBC Devastating Fort McMurray wildfire declared out 15 months later | CBC News. (2017) Available at: http://www.cbc.ca/news/canada/edmonton/fort-mcmurray-fire-beast-extinguished-out-1.4271604 [Accessed April 17, 2018].

[2] Cryderman K Fort McMurray wildfires to cost insurers $3.6-billion, The Globe and Mail. (2016) Available at: https://www.theglobeandmail.com/report-on-business/fort-mcmurray-wildfire-damage-to-cost-36-billion-insurance-bureau/article30788517/ [Accessed May 10, 2018].

[3] Cohan CL Cole SW Life course transitions and natural disaster: marriage, birth, and divorce following Hurricane Hugo. J Fam Psychol (2002) 16:14–25. 10.1037//0893-3200.16.1.14 11915406

[4] Jones RT Ribbe DP Cunningham PB Weddle JD Langley AK Psychological impact of fire disaster on children and their parents. Behav Modif (2002) 26:163–86. 10.1177/0145445502026002003 11961911

[5] Kirsch KR Feldt BA Zane DF Haywood T Jones RW Horney JA Longitudinal community assessment for public health emergency response to wildfire, Bastrop County, Texas. Health Secur (2016) 14:93–104. 10.1089/hs.2015.0060 27081889

[6] Psarros C Theleritis C Economou M Tzavara C Kioulos KT Mantonakis L Insomnia and PTSD one month after wildfires: evidence for an independent role of the “fear of imminent death.” Int J Psychiatry Clin Pract (2017) 21:137–41. 10.1080/13651501.2016.1276192 28084115

[7] Bryant RA Gibbs L Gallagher HC Pattison P Lusher D MacDougall C Longitudinal study of changing psychological outcomes following the Victorian Black Saturday bushfires. Aust N Z J Psychiatry (2017) 52(6):000486741771433. 10.1177/0004867417714337 28605987

[8] Papanikolaou V Adamis D Mellon RC Prodromitis G Psychological distress following wildfires disaster in a rural part of Greece: a case-control population-based study. Int J Emerg Ment Health (2011) 13:11–26. 21957753

[9] Afifi WA Felix ED Afifi TD The impact of uncertainty and communal coping on mental health following natural disasters. Anxiety Stress Coping (2012) 25:329–47. 10.1080/10615806.2011.603048 21801075

[10] Caamano-Isorna F Figueiras A Sastre I Montes-Martínez A Taracido M Piñeiro-Lamas M Respiratory and mental health effects of wildfires: an ecological study in Galician municipalities (north-west Spain). Environ Heal (2011) 10:48. 10.1186/1476-069X-10-48 PMC3224540 21600035

[11] McDermott BM Lee EM Judd M Gibbon P Posttraumatic stress disorder and general psychopathology in children and adolescents following a wildfire disaster. Can J Psychiatry (2005) 50:137–43. 10.1177/070674370505000302 15830823

[12] Papadatou D Giannopoulou I Bitsakou P Bellali T Talias MA Tselepi K Adolescents’ reactions after a wildfire disaster in Greece. J Trauma Stress (2012) 25:57–63. 10.1002/jts.21656 22298431

[13] Goldmann E Galea S Mental health consequences of disasters. Annu Rev Public Health (2014) 35:169–83. 10.1146/annurev-publhealth-032013-182435 24159920

[14] Kar N Psychological impact of disasters on children: review of assessment and interventions. World J Pediatr (2009) 5:5–11. 10.1007/s12519-009-0001-x 19172325

[15] Tang B Liu X Liu Y Xue C Zhang L A meta-analysis of risk factors for depression in adults and children after natural disasters. BMC Public Health (2014) 14:623. 10.1186/1471-2458-14-623 24941890 PMC4077641

[16] Dai W Chen L Lai Z Li Y Wang J Liu A The incidence of post-traumatic stress disorder among survivors after earthquakes: a systematic review and meta-analysis. BMC Psychiatry (2016) 16:188. 10.1186/s12888-016-0891-9 27267874 PMC4895994

[17] Kõlves K Kõlves KE De Leo D Natural disasters and suicidal behaviours: a systematic literature review. J Affect Disord (2013) 146:1–14. 10.1016/j.jad.2012.07.037 22917940

[18] Ungar M The social ecology of resilience: addressing contextual and cultural ambiguity of a nascent construct. Am J Orthopsychiatry (2011) 81:1–17. 10.1111/j.1939-0025.2010.01067.x 21219271

[19] Rutter M Implications of resilience concepts for scientific understanding. Ann N Y Acad Sci (2006) 1094:1–12. 10.1196/annals.1376.002 17347337

[20] Felix E Afifi T Kia-Keating M Brown L Afifi W Reyes G Family functioning and posttraumatic growth among parents and youth following wildfire disasters. Am J Orthopsychiatry (2015) 85:191–200. 10.1037/ort0000054 25822609

[21] Bryant RA Gallagher HC Gibbs L Pattison P MacDougall C Harms L, Mental health and social networks after disaster. Am J Psychiatry (2017) 174:277–85. 10.1176/appi.ajp.2016.15111403 27838935

[22] Brown MRG Agyapong V Greenshaw AJ Cribben I Brett-MacLean P Drolet J After the Fort McMurray wildfire there are significant increases in mental health symptoms in grade 7–12 students compared to controls. BMC Psychiatry (2019) 19:18. 10.1186/s12888-018-2007-1 30630501 PMC6329184

[23] Foa EB Johnson KM Feeny NC Treadwell KR The child PTSD Symptom Scale: a preliminary examination of its psychometric properties. J Clin Child Psychol (2001) 30:376–84. 10.1207/S15374424JCCP3003_9 11501254

[24] Johnson JG Harris ES Spitzer RL Williams JBW The patient health questionnaire for adolescents: validation of an instrument for the assessment of mental disorders among adolescent primary care patients. J Adolesc Health (2002) 30:196–204. 10.1016/S1054-139X(01)00333-0 11869927

[25] Spitzer RL Kroenke K Williams JBW Group and the PHQPCS. Validation and utility of a self-report version of PRIME-MD: the PHQ primary care study. JAMA (1999) 282:1737. 10.1001/jama.282.18.1737 10568646

[26] Zigmond AS Snaith RP The hospital anxiety and depression scale. Acta Psychiatr Scand (1983) 67:361–70. 10.1111/j.1600-0447.1983.tb09716.x 6880820

[27] Knight JR Shrier LA Bravender TD Farrell M Vander Bilt J Shaffer HJ A new brief screen for adolescent substance abuse. Arch Pediatr Adolesc Med (1999) 153:591–6. 10.1001/archpedi.153.6.591 10357299

[28] Knight JR Sherritt L Harris SK Gates EC Chang G Validity of brief alcohol screening tests among adolescents: a comparison of the AUDIT, POSIT, CAGE, and CRAFFT. Alcohol Clin Exp Res (2003) 27:67–73. 10.1111/j.1530-0277.2003.tb02723.x 12544008

[29] Rosenberg M Society and the adolescent self-image. Princeton, NJ: Princeton University Press (1965). 10.1515/9781400876136

[30] Ravens-Sieberer U Erhart M Rajmil L Herdman M Auquier P Bruil J Reliability, construct and criterion validity of the KIDSCREEN-10 score: a short measure for children and adolescents’ well-being and health-related quality of life. Qual Life Res (2010) 19:1487–500. 10.1007/s11136-010-9706-5 PMC2977059 20668950

[31] Liebenberg L Ungar M LeBlanc JC The CYRM-12: a brief measure of resilience. Can J Public Health (2013) 104:e131–5. 10.1007/BF03405676 PMC6974279 23618205

[32] Kroenke K Spitzer RL Williams JB The PHQ-9: validity of a brief depression severity measure. J Gen Intern Med (2001) 16:606–13. 10.1046/j.1525-1497.2001.016009606.x PMC1495268 11556941

[33] Richardson LP McCauley E Grossman DC McCarty CA Richards J Russo JE Evaluation of the Patient Health Questionnaire-9 Item for detecting major depression among adolescents. Pediatrics (2010) 126:1117–23. 10.1542/peds.2010-0852 PMC3217785 21041282

[34] Snaith RP The Hospital Anxiety and Depression Scale. Health Qual Life Outcomes (2003) 1:29. 10.1186/1477-7525-1-29 12914662 PMC183845

[35] Stewart RW Ebesutani C Drescher CF Young J The Child PTSD Symptom Scale: an investigation of its psychometric properties. J Interpers Violence (2015) 32:2237–56. 10.1177/0886260515596536 26270934

[36] 36.ISSTS Child PTSD Symptom Scale. Available at: http://www.istss.org/assessing-trauma/child-ptsd-symptom-scale.aspx [Accessed January 1, 2012].

[37] Hazell P Depression in children and adolescents. BMJ Clin Evid (2009). PMC2907806 19445770

[38] Tramonte L Willms D The prevalence of anxiety among middle and secondary school students in Canada. Can J Public Health (2010) 101 Suppl:S19–22. 10.1007/BF03403977 21416799 PMC6974041

[39] CAMH Drug Use Among Ontario Students, Detailed Findings from the OSDUHS. (2017).

[40] Jones RT Ribbe DP Cunningham P Psychosocial correlates of fire disaster among children and adolescents. J Trauma Stress (1994) 7:117–22. 10.1007/BF02111917 8044435

[41] McLennan J Evans L Cowlishaw S Pamment L Wright L Secondary traumatic stress in postdisaster field research interviewers. J Trauma Stress (2016) 29:101–5. 10.1002/jts.22072 26789530

[42] Alexander DA Klein S First responders after disasters: a review of stress reactions, at-risk, vulnerability, and resilience factors. Prehosp Disaster Med (2009) 24:87–94. 10.1017/S1049023X00006610 19591301

[43] Silverstone PH Bercov M Suen VYM Allen A Cribben I Goodrick J Initial findings from a novel school-based program, EMPATHY, which may help reduce depression and suicidality in youth. PLoS One (2015) 10:e0125527. 10.1371/journal.pone.0125527 25974146 PMC4431804

[44] Silverstone PH Bercov M Suen VYM Allen A Cribben I Goodrick J Long-term results from the empowering a multimodal pathway toward healthy youth program, a multimodal school-based approach, show marked reductions in suicidality, depression, and anxiety in 6,227 students in grades 6–12 (aged 11–18). Front Psychiatry (2017) 8:81. 10.3389/fpsyt.2017.00081 28555115 PMC5430037

